# Prevalence and Characteristics of *Salmonella* from a Laying Hen Production Chain in Jiangsu Province, China

**DOI:** 10.3390/microorganisms14081767

**Published:** 2026-08-11

**Authors:** Aihong Xia, Xin Li, Changjing Zhao, Yaya Wen, Zhaoli Cao, Xubin Lu, Xiaojian Gao, Genxi Zhang, Bo Wang, Yongjuan Wang

**Affiliations:** 1 College of Veterinary Medicine, Jiangsu Agri-Animal Husbandry Vocational College, Taizhou 225300, China; xah@jsahvc.edu.cn (A.X.); zhaochangjing163@163.com (C.Z.); 18865053326@163.com (Y.W.); 2022010490@jsahvc.edu.cn (Z.C.); 2 Anhui Yihua Agriculture and Animal Husbandry Technology Co., Ltd., Chizhou 247100, China; 3 School of Pharmacy, Taizhou University, Taizhou 225300, China; tc_lixin_0318@163.com; 4 College of Animal Science and Technology, Yangzhou University, Yangzhou 225009, China; lxb@yzu.edu.cn (X.L.); gaoxj336@163.com (X.G.); gxzhang@yzu.edu.cn (G.Z.); 5 College of Food Science and Engineering, Yangzhou University, Yangzhou 225009, China

**Keywords:** *Salmonella*, laying hen production chain, resistance, whole-genome sequence analysis, core-genome SNP

## Abstract

Salmonellosis is a significant zoonotic and foodborne disease caused by *Salmonella*. As a primary reservoir, poultry infections can result in decreased survival rates, diminished production performance and contamination of poultry products, thereby posing a serious threat to the sustainable development of poultry farming and human health. In this study, a total of 979 samples were collected from a laying hen production chain, and 84 *Salmonella* strains *were* isolated, with an isolation rate of 8.58% (84/979). Among these, 54 isolates (11.2%) were from commercial house samples, followed by nine isolates (10.8%) from market samples, 10 isolates (5.88%) from hatchery samples, and 11 isolates (4.55%) from breeder house samples. Six different serotypes were identified, with *Salmonella enterica serovar* Enteritidis (*S.* Enteritidis) being the predominant serotype (41.7%), followed by *Salmonella enterica serovar* Typhimurium (*S.* Typhimurium) (22.6%) and *Salmonella enterica serovar* Infantis (*S.* Infantis) (21.4%). The *S.* Enteritidis was distributed in various production stages in the laying hen production chain. Among the 16 antibiotics tested, 30 selected strains exhibited 100% resistance to nalidixic acid (NAL) and ciprofloxacin (CIP), attributed to mutations in the quinolone resistance-determining region (QRDR) of the *gyrA* gene. Following this, the resistance rates for ampicillin (AMP), amoxicillin–clavulanate (AMC), and streptomycin (STR) were 56.7%. Antimicrobial resistance genotypes were consistent with the drug resistance phenotype. Whole genome sequencing (WGS) phylogenetic analysis based on core genome single nucleotide polymorphism (cgSNP) revealed that *Salmonella* can spread across various stages of the laying hen production chain, posing a potential threat to human health. Furthermore, 165 virulence-associated genes were identified among the investigated isolates, indicating a highly conserved virulence repertoire. This study investigates the prevalence of *Salmonella* within a laying hen production chain, providing a scientific foundation for the effective prevention and control of *Salmonella* infection.

## 1. Introduction

*Salmonella* belongs to the Enterobacteriaceae family and comprises two species: *Salmonella bongori* (*S*. *bongori*) and *Salmonella* enterica (*S*. *enterica*). The species *S*. *enterica* includes six subspecies, with a total of approximately 2600 serotypes [1]. In poultry, infections caused by *Salmonella enterica serovar* Pullorum (*S*. Pullorum) and *Salmonella enterica serovar* Gallinarum (*S*. Gallinarum) result in pullorum disease and fowl typhoid, respectively, whereas infections caused by numerous other non-host-adapted serovars are termed paratyphoid [2]. Unlike pullorum disease and fowl typhoid, paratyphoid infections are usually asymptomatic or subclinical in poultry, allowing infected poultry to act as persistent carriers and continuously shed *Salmonella* into the farm environment [3]. Consequently, poultry and poultry products represent important reservoirs and vehicles for the transmission of *Salmonella* to humans. Among the numerous serovars associated with poultry, *Salmonella enterica serovar* Enteritidis (*S*. Enteritidis), *Salmonella enterica serovar* Typhimurium (*S*. Typhimurium), *Salmonella enterica serovar* Infantis (*S*. Infantis), and *Salmonella enterica serovar* Kentucky (*S*. Kentucky) are among the most frequently reported and are of major public health concern because of their high prevalence, antimicrobial resistance, and ability to cause foodborne infections [4,5].

Egg, as a high-protein, low-fat, multi-nutrient food, has become an important part of a healthy dietary pattern [6] and the consumption of eggs is increasing year by year worldwide [7]. According to statistics, over a billion cases of gastroenteritis caused by *Salmonella* are reported worldwide annually [8]. Each year in the United States, there are one million cases of foodborne illness related to non-typhoid *Salmonella*, specifically *S*. Enteritidis, which leads to human salmonellosis mainly through contaminated eggs or egg products [9]. Between 2015 and 2018, a total of 1209 large-scale infections caused by *S.* Enteritidis-contaminated eggs were reported in 16 European countries [10]. In China, a total of 55,266 non-typhoidal *Salmonella* (NTS) infections were reported through the national foodborne disease surveillance system during 2013–2022, and the reporting rate of NTS was strongly correlated with the national per-capita consumption of eggs [11]. In the poultry production chain, *Salmonella* can be present at various stages of production, ultimately contaminating the entire chain through both vertical and horizontal transmission [12]. Lei et al. [13] demonstrated that 148 strains of *Salmonella* were isolated from 2100 fecal swab samples, and the isolation rate of *Salmonella* was 5.3% at the breeder house, 4.2% at the hatchery, and 10.4% at the commercial chicken farm. Liu et al. [14] conducted continuous monitoring of *Salmonella* on a large-scale intensive laying hen farm from 2020 to 2023, isolating 105 strains from 1537 samples, and the commercial areas were identified as the most contaminated with *Salmonella*. Therefore, comprehensive prevention and control measures should be implemented across the entire farming process to mitigate the risk of contamination.

Recent years have witnessed substantial advancements in the molecular typing analysis of foodborne pathogens with whole-genome sequencing (WGS). WGS has become an effective tool for investigating serotyping, antimicrobial resistance genes, pathogenicity characteristics and the source of *Salmonella*. Some researchers [15] employed WGS to study phylogenetic relationships of *Salmonella* at different stages of chicken slaughter in East China, and the slaughter environment was recognized as the source of cross-contamination during processing. Liu et al. [14] conducted a source-trace analysis of *S.* Kentucky from a laying hen farm using WGS in conjunction with epidemiological investigations, revealing that *S*. Kentucky entered the farm via an introduction link.

In this study, we conducted a surveillance study to assess the prevalence of *Salmonella* within a laying hen production chain. Samples were collected from a breeder house and hatchery in Yangzhou, Jiangsu Province, as well as from a commercial house and market in Taizhou, Jiangsu Province. Subsequently, the antimicrobial resistance profiles of *Salmonella* isolates were investigated to provide a scientific basis for optimizing production management models. Furthermore, WGS was performed to characterize their antimicrobial resistance genes, phylogenetic relationships and virulence genes. The purpose of these analyses was to collect data and promote the prevention and control of *Salmonella* contamination in this laying hen production chain.

## 2. Materials and Methods

### 2.1. Sample Collection

The sampling strategy was designed to comprehensively evaluate *Salmonella* contamination across the laying hen production chain. Parent breeder chickens are raised in the breeder house for egg production, and the eggs are subsequently hatched. Once the chicks reach a certain age, they are transferred to the commercial house for egg laying. Eggs were then transported to the market for sale. Therefore, sampling sites were selected to cover four critical stages of the production chain, including the breeder house, the hatchery, the commercial house and the market, which represent potential points of *Salmonella* introduction, persistence and transmission. Different sample types, including sick or dead chicken, dead embryos, environment, feces, feed, water and eggs, were collected to assess contamination sources. Environmental samples consisted of swabs collected from egg belts, manure belts, ventilation fans, floors, workers’ boot soles, and egg trays, as appropriate for each sampling site. The sample size was determined based on the availability of representative sampling sites, production characteristics, and the need to cover different stages of the production system.

**Figure 1:**
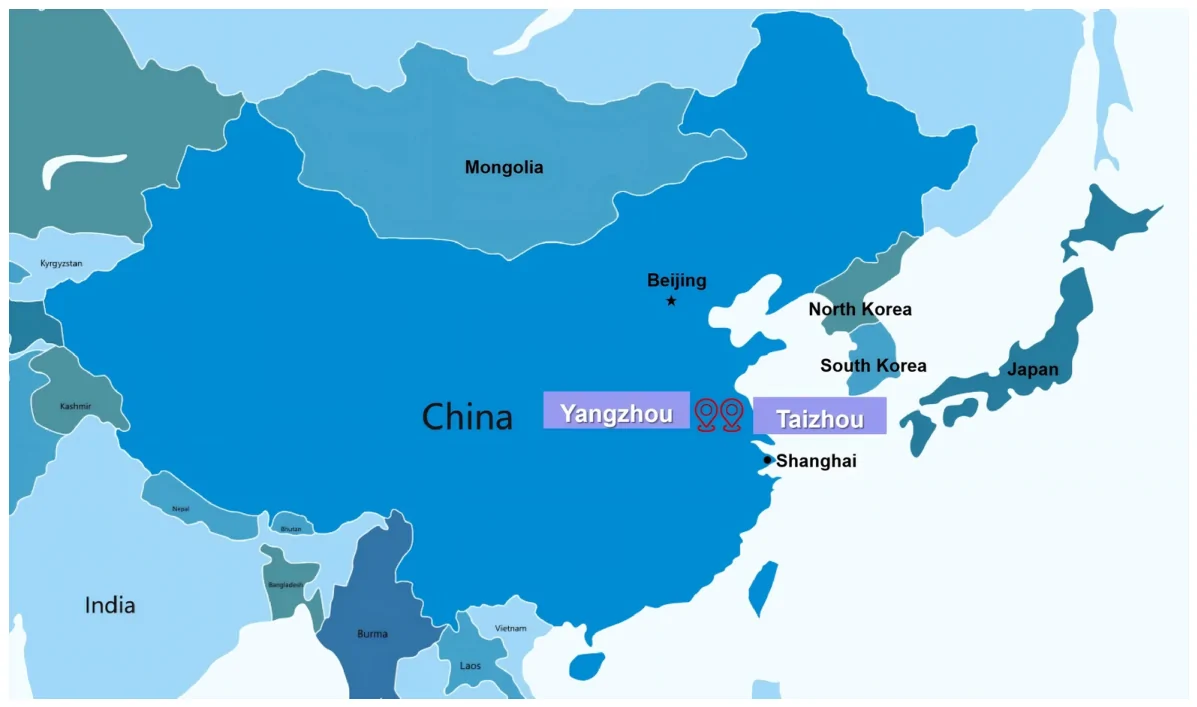
Geographical locations of Yangzhou and Taizhou in Jiangsu Province, China.

Samples were collected from different stages of the laying hen production chain, which includes one breeder house and one hatchery in Yangzhou, Jiangsu Province, as well as one commercial house (a family farm) and one market in Taizhou, Jiangsu Province, for *Salmonella* isolation (Figure 1). All samples were stored on ice and transported to the laboratory within 24 h. Upon arrival at the laboratory, the samples were processed immediately. Liver samples from necropsied sick or dead chicken and yolk sac samples from dead embryos were aseptically collected for *Salmonella* isolation.

### 2.2. Isolation and Identification of Salmonella

The buffered peptone water (BPW) (Haibo, Qingdao, China) was added to each sample at a volume ratio of 1:10 relative to the sample volume, followed by incubation at 37 °C for 16–20 h for preliminary enrichment. Then, 1 mL of preliminary enrichment culture was added to 10 mL of Rappaport-Vassiliadis R10 (RvR10) broth (Haibo) and incubated at 42 °C for 24 h [14,16]. Upon completion of incubation, selective enrichment culture was streaked onto xylose lysine tergitol 4 (XLT4) agar plates (Haibo) and incubated at 37 °C for 24 h. Polymerase chain reaction (PCR) was employed to determine whether the isolates were *Salmonella* spp. using specific primers for *stn* amplification [17]. The serotypes of the isolates were determined by slide agglutination using commercial *Salmonella* O and H antisera (Tianrun, Ningbo, China) according to the Kauffmann–White scheme.

### 2.3. Antimicrobial Susceptibility Testing

According to the Clinical and Laboratory Standards Institute (CLSI 2021) [18], the antimicrobial susceptibility of representative *Salmonella* isolates was tested with the disk diffusion method. In brief, a single colony was inoculated into 4 mL of MH liquid medium and incubated at 37 °C for 16–18 h. The bacterial suspension was calibrated to 0.5 McFarland units with sterile PBS solution and uniformly distributed onto MH solid agar plates. Antibiotic discs were placed on the infected MH solid agar surface with a disc dispenser. Plates were incubated at 37 °C for 24 h, after which the diameters of the zones were measured and compared to standard criteria. A total of 16 antimicrobial agents were used: ampicillin (AMP), amoxicillin–clavulanate (AMC), cefazolin (CFZ), cefotaxime (CTX), aztreonam (ATM), meropenem (MEM), gentamicin (GEN), amikacin (AK), streptomycin (STR), enrofloxacin (ENR), nalidixic acid (NAL), ciprofloxacin (CIP), tetracycline (TET), trimethoprim–sulfamethoxazole (SXT), chloramphenicol (CHL), and nitrofurantoin (F). *Escherichia coli* ATCC 25922 served as a control strain. Multidrug-resistant (MDR) and extensively drug-resistant (XDR)bacteria were defined according to the international criteria proposed by Magiorakos et al. [19].

### 2.4. WGS

WGS was performed on a total of 42 *Salmonella* isolates, including 35 *S*. Enteritidis isolates and 7 *S*. Kentucky isolates collected from the laying hen production chain. Antimicrobial resistance gene analysis was performed using the WGS data generated from the same 30 isolates used for antimicrobial susceptibility testing, including 23 *S*. Enteritidis isolates and all 7 *S*. Kentucky isolates, to evaluate the relationship between antimicrobial resistance genotypes and phenotypic resistance profiles. The 35 *S*. Enteritidis isolates recovered from different stages of the laying hen production chain were subjected to genomic epidemiological analyses, including MLST, phylogenetic analysis, and virulence gene characterization.

The genomic DNA of the isolated strains was extracted using the TIAN amp Bacteria DNA Kit (Tiangen, Beijing, China) according to the manufacturer’s instructions. All genomes were fragmented to an insertion size of 500 bp for library construction. WGS was conducted using the Illumina NovaSeq 6000 platform (Illumina, San Diego, CA, USA), producing 150 bp paired-end reads. Genomes were de novo assembled using SPAdes 3.15.5. Multilocus sequence typing (MLST), antimicrobial resistance genes, core genome SNP and virulence gene analysis were performed according to the methods described by Kang et al. [16] and Jin et al. [20]. Serotypes were also predicted from the WGS data using SeqSero2 and compared with those determined by conventional serological agglutination. WGS data of all *Salmonella* isolates were submitted to the NCBI database with the accession number PRJNA1479310.

### 2.5. Statistical Analysis

Data of *Salmonella* isolation prevalence was analyzed using SPSS 22.0 software. The chi-square test was employed to assess the significance of differences, with *p* < 0.05 indicating significant differences.

## 3. Results

### 3.1. Prevalence of Salmonella in a Laying Hen Production Chain

A total of 979 samples were collected from different stages of the laying hen production chain in Jiangsu Province, including a breeder house, a hatchery, a commercial house, and a market (Table 1). Among these samples, 242 samples were collected from the breeder house, which included samples from sick or dead chickens, environment, feces, feed and water. A total of 170 samples were collected from the hatchery, including dead embryo samples and environment samples. In addition, 484 samples were collected from the commercial house, which included samples from sick or dead chickens, environment, feces, eggs, feed and water. The remaining 83 samples were collected from markets, including egg samples and environmental samples. Among the 979 samples collected, 84 *Salmonella* isolates were recovered, yielding an overall prevalence rate of 8.58%. The highest isolation rate was observed in the commercial house (11.2%), followed by markets (10.8%). In contrast, the prevalence in the breeder house and the hatchery was 4.6% and 5.9%, respectively. The isolation rate in the commercial house was significantly higher than that in the breeder house and the hatchery (*p* < 0.05). Environmental samples from the commercial house and markets exhibited relatively high isolation rates. The isolation rate of environmental samples reached 21.4% in markets and 17.3% in the commercial house. Among egg samples, the highest prevalence was detected in markets (5.5%). *Salmonella* was not detected in any feed or water samples.

### 3.2. Serotyping of Salmonella Isolates

**Figure 2:**
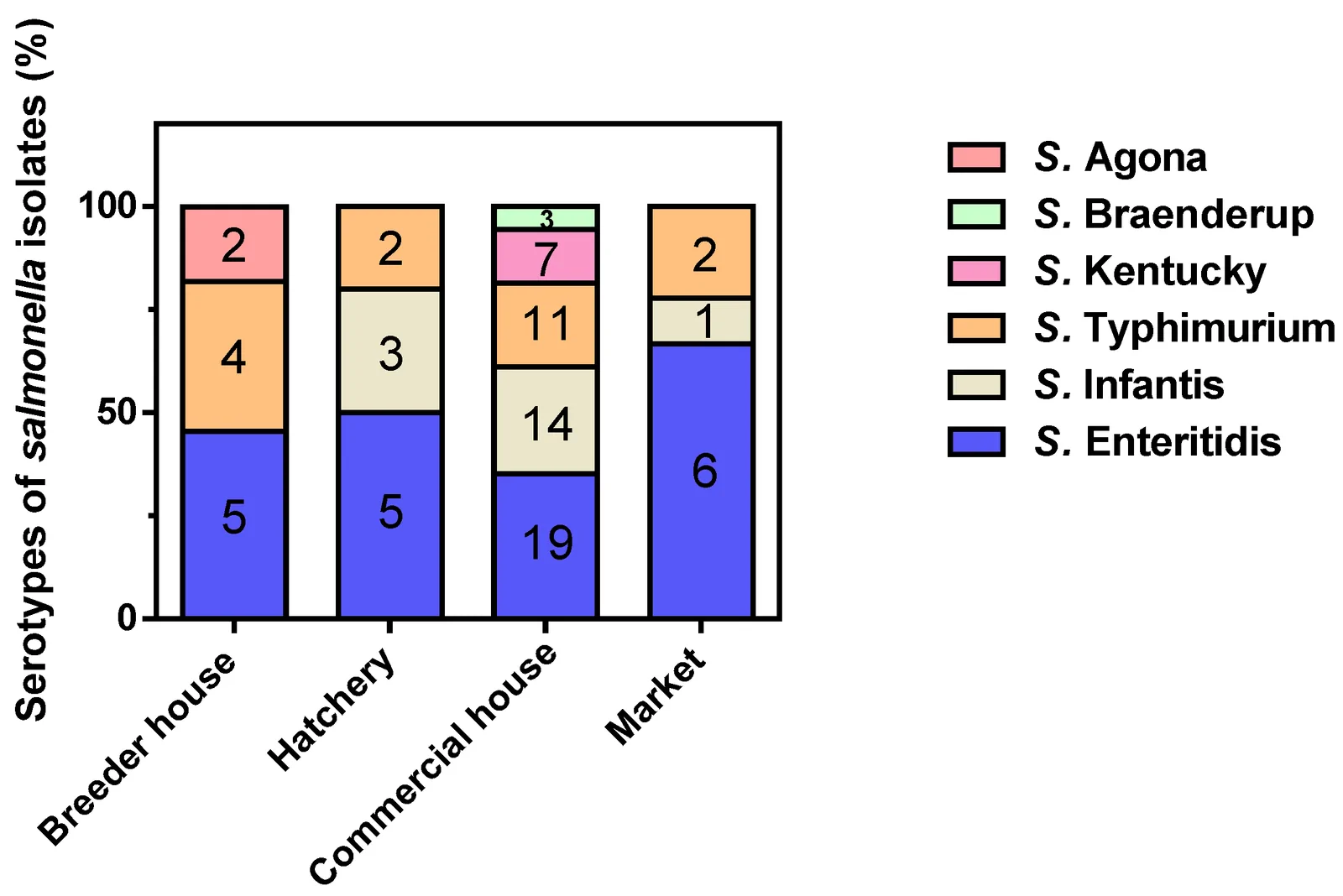
Serotype distribution of *Salmonella* isolates recovered from different stages of the laying hen production chain. Data are presented as the number of isolates for each serotype across different sampling stages.

Serotyping of the 84 *Salmonella* isolates identified six distinct serovars. *S.* Enteritidis was the predominant serovar, accounting for 41.7% of the isolates, followed by *S*. Typhimurium (22.6%) and *S*. Infantis (21.4%) (Figure 2). In addition, seven isolates were identified as *S*. Kentucky, three as *Salmonella enterica serovar* Brenderup (*S*. Brenderup), and two as *Salmonella enterica serovar* Agona (*S*. Agona). *S.* Enteritidis was the dominant serovar throughout the entire laying hen production chain and was detected in the breeder house, hatchery, commercial house, and markets. Notably, SeqSero2-based serovar predictions were fully concordant with conventional serological agglutination results among all WGS-sequenced isolates, showing 100% agreement between the two approaches.

### 3.3. Antimicrobial Resistance Phenotypes and Genotypes

Following serotyping, antimicrobial susceptibility testing of 30 representative isolates was performed. Among the 35 *S*. Enteritidis isolates identified in this study, 23 isolates were selected for antimicrobial susceptibility testing. These isolates were recovered from different stages of the laying hen production chain and represented the circulating *S*. Enteritidis isolates across the production chain. In addition, all seven *S*. Kentucky isolates recovered from the commercial house were included because of their highly resistant phenotype (Table 2). Among the tested isolates, 56.7% exhibited a MDR phenotype. Notably, all *S*. Kentucky isolates recovered from the commercial house were resistant to antimicrobial agents belonging to all but two antimicrobial categories, representing an XDR phenotype. The highest resistance rates were observed for NAL and CIP (both 100%), followed by AMP, AMC, and STR (56.7%). Resistance to TET was 53.3%, and 33.3% of the isolates were resistant to ENR. Resistance to the remaining antibiotics was primarily detected in *S*. Kentucky isolates. When analyzed by different stages of the production chain, *S*. Enteritidis isolates from the breeder house and hatchery exhibited 100% resistance to NAL and CIP and 30% resistance to ENR, while remaining susceptible to the other 13 tested antibiotics. In contrast, *S*. Enteritidis isolates from the commercial house and markets showed 100% resistance to NAL and CIP and 76.9% resistance to AMP, AMC, and STR. Additionally, these isolates demonstrated resistance to CFZ (7.7%), TET (69.2%), and F (10.5%).

WGS was subsequently performed to detect antimicrobial resistance genes among those 30 isolates (Table 3). All isolates harbored the aminoglycoside resistance gene *aac(6′)-Iaa*. The detection rates of *aph(3″)-Ib*, *aph(6)-Id* (aminoglycoside resistance) and *sul2* (sulfonamide resistance) were both 33.33%, while *tet(A)* (tetracycline resistance) and *bla*_TEM-1B_ (β-lactam resistance) were detected in 53.33% and 56.67% of isolates, respectively. The resistance genes *aph(3′)-Ia*, *aadA7*, *rmtB*, *sul1*, *bla*_CTX-M-55_, *qnrS1*, *dfrA14*, *lnu(F)*, *ARR-2*, *floR*, *mph(A)* and *fosA3* were exclusively identified in the seven *S.* Kentucky isolates.

The detected antimicrobial resistance genes were generally consistent with the antimicrobial resistance profiles (Supplementary Table S1). The aminoglycoside acetyltransferase gene *aac(6′)-Iaa* was identified in all 30 *Salmonella* isolates. However, isolates carrying only *aac(6′)-Iaa* remained susceptible to GEN and AMI, indicating that the presence of *aac(6′)-Iaa* alone did not confer phenotypic resistance to these aminoglycosides [21]. The aminoglycoside resistance genes *aph(3″)-Ib*, *aph(6)-Id* and *aadA7* were associated with STR resistance, whereas the tetracycline resistance gene *tet(A)* was identified in all TET-resistant isolates. The β-lactamase gene *bla*_TEM-1B_ was detected in all AMP-resistant isolates. Resistance to AMC was also observed in those isolates, suggesting that additional resistance mechanisms may contribute to the AMC-resistant phenotype. Isolates carrying *sul2* alone remained susceptible to SXT, whereas those co-harboring *sul1* and *dfrA14* exhibited SXT resistance. All isolates were resistant to NAL and CIP. WGS identified mutations within the QRDR of the gyrA gene, including Asp87Tyr (D87Y) in 33.3% (10/30) of isolates, Asp87Asn (D87N) in 43.3% (13/30), and combined Ser83Phe (S83F) and Asp87Asn (D87N) substitutions in the remaining 23.3% (7/30). Furthermore, the plasmid-mediated quinolone resistance gene *qnrS1* was detected exclusively in the seven *S*. Kentucky isolates. These findings indicate that QRDR mutations in *gyrA*, together with plasmid-mediated quinolone resistance determinants, were associated with the universal quinolone-resistant phenotype observed in this study. Apart from the resistance determinants described above, all remaining acquired resistance genes were found only in the seven XDR *S*. Kentucky isolates, and their presence was generally consistent with the observed antimicrobial resistance phenotypes.

### 3.4. Traceability Analysis of S. Enteritidis

**Figure 3:**
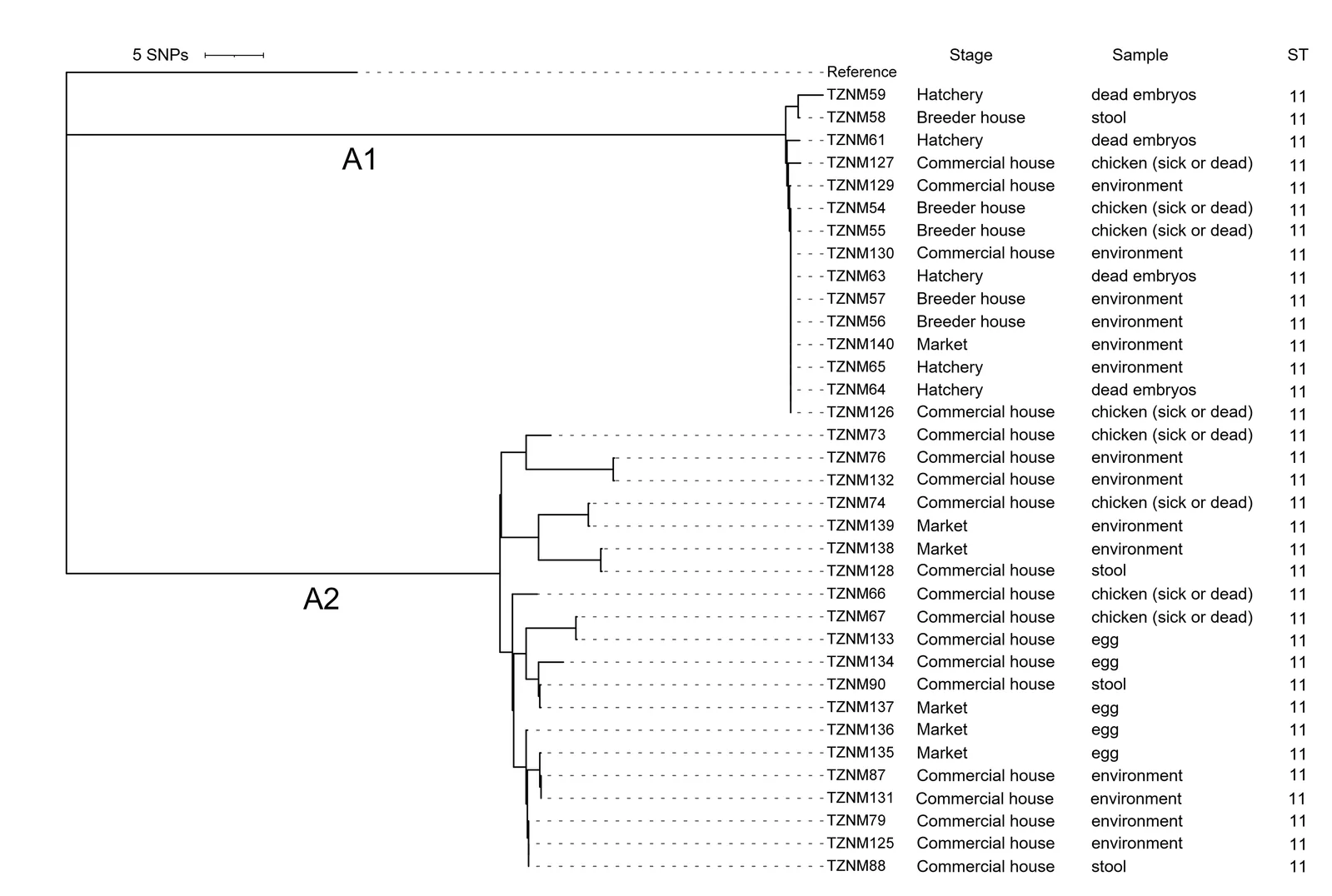
Phylogenetic tree of *S*. Enteritidis isolates based on core-genome SNPs. The analysis included 35 *S*. Enteritidis isolates from different laying hen production stages.

WGS was performed on 35 *S*. Enteritidis isolates. Multilocus sequence typing (MLST) analysis revealed that all isolates belonged to sequence type 11 (ST11) (Figure 3). An evolutionary tree was constructed based on core genome single nucleotide polymorphism (cgSNP), and the isolates were divided into two distinct clades (A1 and A2). Clade A1 included strains recovered from the breeder house, hatchery, commercial house and markets. These isolates shared high genetic relatedness (the pairwise SNP distance values < 10) and belonged to the same clonal cluster, suggesting that this clone may disseminate along the egg production chain.

### 3.5. Virulence Genes Analysis

The genomes of all 35 *S*. Enteritidis isolates were screened against the Virulence Factor Database (VFDB) to identify virulence-associated genes involved in adhesion, invasion, intracellular survival, secretion systems, iron acquisition, motility, environmental adaptation, and other pathogenicity-related functions. The results revealed a highly conserved virulence gene repertoire among the isolates (Supplementary Figure S1). A total of 165 virulence-associated genes were identified, of which 164 were detected in all 35 isolates. These conserved genes included those involved in fimbrial adhesion (including *csg*, *fim*, *lpf* and *pef* operons), flagellar assembly and chemotaxis (*flg*, *flh*, *fli* and *che*), type III secretion systems (T3SSs) (*sip*, *sop*, *ssa*, *ssc*, *sse* and *ssr*), macrophage survival (*mgtC* and *sodCI*), and other virulence-associated functions related to intracellular survival and host colonization. The only variable virulence gene was *shdA*, which was detected in two isolates (TZNM88 and TZNM90) but was absent from the remaining 33 isolates. Overall, the virulence gene profiles were nearly identical across all *S*. Enteritidis isolates, indicating a highly conserved pathogenic potential.

## 4. Discussion

Although the prevalence of *Salmonella* in poultry has been extensively investigated in China [22,23], epidemiological studies focusing on the laying hen production chain remain limited. This study provides a comprehensive epidemiological analysis of *Salmonella* contamination across a laying hen production chain in Jiangsu Province, China, revealing an overall isolation rate of 8.58%, which was higher than prevalence estimates reported in the United States (1.19%) [24] and Ethiopia (4.7%) [25], but is comparable to rates reported from some poultry farms in China, including 6.83% [14] and 10.77% [26]. The isolation rates of *Salmonella* in the breeder house and hatchery were 4.6% and 5.9%, respectively, which are consistent with the relatively low prevalence reported for these upstream production stages in previous studies [27,28]. The relatively lower prevalence in the breeder house and the hatchery may be attributed to stricter biosecurity measures implemented in these upstream stages. The higher prevalence in the commercial house and markets compared to the breeder house and hatchery aligns with findings from other studies, where commercial and downstream stages often show elevated contamination due to increased environmental exposure, multi-age management, and potential cross-contamination [14,26]. Notably, environmental samples from the commercial house and markets in the present study exhibited relatively high isolation rates (17.3% and 21.4%), which is higher than the 7.1% found by Elmonir et al. [29]. This discrepancy may be attributable to differences in production systems, biosecurity practices, sampling strategies, and environmental management [30]. Collectively, these findings suggest that the downstream stages of the laying hen production chain represent critical control points for *Salmonella* contamination. Therefore, enhanced hygiene management, continuous environmental monitoring, and targeted intervention measures at the commercial house and markets are essential to interrupt pathogen dissemination, reduce egg contamination, and ultimately minimize the public health burden associated with foodborne salmonellosis.

Serotyping analysis identified six distinct *Salmonella* serovars among the 84 isolates, with *S*. Enteritidis being the predominant serovar (41.7%) and *S*. Typhimurium and *S*. Infantis being the most widely distributed in the laying hen production. This serotype distribution is consistent with global and regional trends, where *S*. Enteritidis has long been recognized as the leading cause of egg-associated salmonellosis worldwide [31,32]. The U.S. Centers for Disease Control and Prevention (CDC) has repeatedly documented *S*. Enteritidis as a primary serovar in egg-linked outbreaks in the United States [33,34]. It is estimated that approximately 3.25 million eggs in the United States are contaminated with *S*. enteritidis each year [35]. Similarly, the European Food Safety Authority (EFSA) and European Centre for Disease Prevention and Control (ECDC) have reported numerous multi-country outbreaks of *S*. Enteritidis linked to eggs and egg products, including prolonged outbreaks affecting up to 18 countries between 2015 and 2020 [10,36,37]. *S*. Typhimurium was the second most prevalent serovar, accounting for 22.6% of all isolates. This serovar is recognized as one of the leading causes of non-typhoidal salmonellosis worldwide and has been widely isolated from poultry, livestock, food products, and human clinical cases [38]. Owing to its broad host range and zoonotic potential, *S*. Typhimurium represents a major public health concern and continues to impose a substantial burden on both livestock production and human health [39]. Our study also found that *S*. Infantis accounted for 21.4% of the isolates, indicating a notable presence that is consistent with its reported emergence in Chinese poultry production. Recent surveillance in China has shown *S*. Infantis rising in prevalence within poultry and egg-related chains, often ranking among the top serovars alongside *S*. Enteritidis [23,26]. The rapid spread of *S*. Infantis is of particular concern because this serovar has been frequently associated with multidrug resistance and enhanced virulence potential in both poultry and humans [40]. Furthermore, *S*. Kentucky was detected exclusively in the commercial house, and all isolates exhibited an XDR phenotype. Recent studies have demonstrated that the globally disseminated *S*. Kentucky ST198 lineage has become an emerging high-risk clone because of its extensive antimicrobial resistance, widespread circulation across animals, food, and humans, and increasing association with human infections [41,42]. The detection of *S*. Kentucky in the present study therefore raises concerns regarding the potential dissemination of clinically relevant resistance determinants through the laying hen production chain. Notably, the family-operated commercial house harbored the greatest serovar diversity, suggesting that small-scale, open production systems may be more susceptible to the introduction of *Salmonella* from external sources, including wildlife, rodents, farm personnel, and contaminated environments [43,44]. Although *S*. Typhimurium and *S*. Infantis were also isolated in this study, epidemiological analyses focused on the predominant serovar, *S*. Enteritidis, and the XDR *S*. Kentucky isolates. Future investigations including these additional serovars would improve our understanding of the molecular epidemiology and transmission dynamics of *Salmonella* in laying hen production chains.

Antimicrobial resistance (AMR) is a major global public health threat, and the emergence of multidrug-resistant *Salmonella* in poultry production has significantly complicated the treatment of salmonellosis [45]. In this study, approximately 60% of the 30 tested *Salmonella* isolates exhibited multidrug resistance. Resistance was most frequently observed to quinolones, followed by β-lactams, streptomycin, and tetracyclines. This resistance profile is consistent with those reported in recent studies of poultry-derived *Salmonella* [46,47]. NAL has not been recently applied to control bacterial contamination. Hence, the emergence of drug resistance can be largely explained by the historical use of quinolone antibiotics. The increasing prevalence of NAL resistance raises major concerns, as it may impair the efficacy of fluoroquinolone treatments and ultimately result in delayed or failed therapy [48]. *Salmonella* isolates from the commercial house and market displayed broader resistance spectra than those from the breeder house and hatchery, which may reflect long-term antimicrobial selection pressure during commercial production and environmental exposure. In particular, *S*. Kentucky isolated from the commercial house exhibited resistance to 15 distinct antibiotics, representing a typical XDR phenotype. XDR *S*. Kentucky strains have been regarded as a serious global public health concern in recent years [42,49], with spread in poultry and humans. WGS revealed that the 30 isolates used for antimicrobial susceptibility testing harbored a diverse repertoire of antimicrobial resistance genes, including the commonly reported *aadA7*, *aph(3′)-Ia*, and *aph(3″)-Ib*; *tet(A), sul1*, and *sul2*; as well as some high prevalence rate common genes such as *bla*_CTX-M-55_, *bla*_TEM-1B_, *floR*, *qnrS1*, and *mph(A)* [50,51,52]. Overall, the antimicrobial resistance genotypes showed a high degree of concordance with the corresponding phenotypic resistance profiles. Specifically, *bla*_TEM-1B_ was strongly associated with AMP resistance [53]. Likewise, *tet(A)* was strongly associated with TET resistance [21]; *aph(3″)-Ib*, *aph(6)-Id* and *aadA7* were associated with STR resistance [54]; and resistance to SXT was often observed in isolates co-harboring *sul1* and *dfrA14* [49]. Quinolone resistance was primarily mediated by mutations in the quinolone resistance-determining region (QRDR) of the *gyrA* gene. Single mutations at codons 83 or 87 of *gyrA* typically confer high-level resistance to nalidixic acid, while dual mutations significantly elevate resistance to ciprofloxacin and other fluoroquinolones [55,56]. Characterization of antimicrobial resistance determinants in poultry-associated *Salmonella* is essential for understanding the evolution and dissemination of antimicrobial resistance, guiding targeted intervention strategies, and mitigating the growing public health threat posed by antimicrobial-resistant *Salmonella*.

Studies have shown that WGS is a powerful tool for the molecular characterization and phylogenetic analysis of *Salmonella* spp. [26]. WGS-based phylogenetic analysis demonstrated that all *S*. Enteritidis isolates belonged to ST11, which is recognized as one of the dominant epidemic sequence types associated with poultry and human infections worldwide [4,57]. Phylogenetic analysis showed that all *S*. Enteritidis isolates were grouped into two major clonal lineages (A1 and A2). Within clade A1, isolates recovered from sick or dead chickens in the commercial house showed close genetic relatedness to isolates obtained from dead embryos and parental sick or dead chickens, providing genomic evidence consistent with vertical transmission of *S*. Enteritidis within the laying hen production chain. Furthermore, genetically closely related isolates recovered from different stages of the laying hen production chain clustered within the same clonal lineage, suggesting that *S*. Enteritidis can disseminate progressively along the production chain and eventually reach downstream markets, thereby posing a potential risk to public health. Because the breeder house, hatchery, commercial house, and market investigated in this study were geographically separated, while isolates recovered from different production stages exhibited high genetic relatedness, our findings support the dissemination of *S*. Enteritidis across geographical regions along the laying hen production chain. Similar observations were reported by Li et al., who demonstrated that *S*. Enteritidis can spread over long distances through poultry breeding hierarchies and breeding-stock trade networks [58]. Isolates within clade A2 exhibited high genomic similarity between the commercial house and the market, indicating the introduction or dissemination of an additional clonal lineage of *S*. Enteritidis into the production chain. As all isolates belonging to this clade were recovered exclusively from the commercial house and market, it is plausible that this clone was introduced into the market during egg transportation via contaminated eggshell surfaces, egg trays, transport equipment, or personnel. Therefore, effective control of *Salmonella* contamination in eggs should not rely solely on interventions at downstream markets but should also include targeted control measures at upstream stages to interrupt the dissemination of *Salmonella* throughout the production chain.

WGS allows researchers to map virulence genes on a larger scale, providing a deeper understanding of how *S. Enteritidis* adapts and persists in specific hosts and environments [35]. The present study revealed a highly conserved virulence profile among *S*. Enteritidis isolates, with most virulence genes detected in all or nearly all isolates, consistent with previous reports [59,60]. These conserved determinants included genes involved in fimbrial adhesion, flagellar motility, type III secretion systems, intracellular survival, macrophage persistence, and iron acquisition, which are known to contribute to host colonization and systemic infection [61]. The high degree of conservation observed in these virulence determinants suggests that the investigated isolates possess comparable pathogenic potential and is consistent with previous reports indicating that *S*. Enteritidis maintain highly stable virulence gene repertoires despite geographical separation [59]. Interestingly, *shdA* was the only variable virulence gene identified among the isolates. Although the biological significance of this variation remains unclear, *shdA* has been associated with intestinal colonization, long-term persistence and fecal shedding in *Salmonella* [62]. Further studies are warranted to determine whether the limited variation in *shdA* influences host adaptation, persistence, or transmission dynamics within poultry production systems.

This study has several limitations. Although samples were collected across multiple stages of the laying hen production chain, certain sample categories had relatively small sample sizes, which may limit the interpretation of contamination patterns for specific sample types. In addition, this study was conducted within the selected laying hen production chain in Jiangsu Province, and therefore the findings may not fully represent the epidemiological status of *Salmonella* contamination in the broader poultry industry. The results should be considered exploratory and provide information regarding the distribution, antimicrobial resistance, and genomic characteristics of *Salmonella* in the investigated laying hen production chains. Future studies involving larger sample sizes and expanded geographic coverage are warranted to further validate these findings.

## 5. Conclusions

This study demonstrated that *Salmonella*, especially *S*. Enteritidis, is widely distributed throughout the investigated laying hen production chain in Jiangsu Province, China and can disseminate among different production stages. The high prevalence of quinolone resistance and the emergence of multidrug-resistant *S*. Kentucky further underline the potential public health risks associated with antimicrobial-resistant *Salmonella* in poultry production. These findings provide important epidemiological evidence supporting the implementation of comprehensive surveillance, strict biosecurity measures, and rational antimicrobial use across the entire egg production chain.

## Figures and Tables

**Table 1:** Isolation of *Salmonella* from different laying hen production stages.

Location	Stage	Sample	Number of Samples	Isolate (%)	Stage Separation Rate (%)
Yangzhou	Breeder house	chicken (sick or dead)	39	2 (5.1)	11/242 (4.6)
		environment	143	6 (4.2)	
		feces	45	3 (6.7)	
		feed	10	0	
		water	5	0	
	Hatchery	dead embryos	115	5 (4.3)	10/170 (5.9)
		environment	55	5 (5.9)	
Taizhou	Commercial house	chicken (sick or dead)	56	9 (16.1)	54/484 (11.2) ^a^
		environment	202	35 (17.3)	
		feces	53	8 (15.1)	
		egg	158	2 (1.3)	
		feed	10	0	
		water	5	0	
	Markets	egg	55	3 (5.5)	9/83 (10.8)
		environment	28	6 (21.4)	
	Total		979	84 (8.6)	

^a^ Indicates a significant difference in commercial house (11.2%) to breeder house (4.6%) or hatchery (5.9%) (*p* < 0.05).

**Table 2:** Antimicrobial resistance phenotypes of 30 *Salmonella* isolates.

	Resistance Number of Different Sample Isolates (%)			
Antibiotic	Breeder House + Hatchery	Commercial House + Market		Total (*n* = 30)
	*S*. Enteritidis (*n* = 10)	*S*. Enteritidis (*n* = 13)	*S*. Kentucky (*n* = 7)	
β-Lactams				
Ampicillin (AMP)	0	10 (76.9)	7 (100)	17 (56.7)
Amoxicillin–clavulanate (AMC)	0	10 (76.9)	7 (100)	17 (56.7)
Cefazolin (CFZ)	0	1 (7.7)	7 (100)	8 (26.7)
Cefotaxime(CTX)	0	0	7 (100)	7 (23.3)
Aztreonam (ATM)	0	0	7 (100)	7 (23.3)
Meropenem (MEM)	0	0	0	0
Aminoglycosides				
Gentamicin (GEN)	0	0	7 (100)	7 (23.3)
Amikacin (AK)	0	0	6 (85.7)	6 (20)
Streptomycin (STR)	0	10 (76.9)	7 (100)	17 (56.7)
Quinolone				
Enrofloxacin (ENR)	3 (30)	0	7 (100)	10 (33.3)
Nalidixic acid (NAL)	10 (100)	13 (100)	7 (100)	30 (100)
Ciprofloxacin(CIP)	10 (100)	13 (100)	7 (100)	30 (100)
Sulfonamides				
Tetracycline (TET)	0	9 (69.2)	7 (100)	16 (53.3)
Trimethoprim–sulfamethoxazole (SXT)	0	0	7 (100)	7 (23.3)
Chloramphenicol (CHL)	0	0	7 (100)	7 (23.3)
Nitrofurantoin (F)	0	2 (10.5)	1 (14.3)	3 (10)

**Table 3:** Antimicrobial resistance genes of 30 *Salmonella* isolates.

Resistance Gene		%
Aminoglycoside resistance	*aac(6′)-Iaa*	100.00
	*aph(3′)-Ia*	23.33
	*aph(3″)-Ib*	33.33
	*aph(6)-Id*	33.33
	*aadA7*	23.33
	*rmtB*	23.33
Sulphonamide resistance	*sul1*	23.33
	*sul2*	33.33
Tetracycline resistance	*tet(A)*	53.33
Beta-lactam resistance	*bla*_CTX-M-55_	23.33
	*bla*_TEM-1B_	56.67
Quinolone resistance	*qnrS1*	23.33
Trimethoprim resistance	*dfrA14*	23.33
Lincosamide resistance	*lnu(F)*	23.33
Rifampicin resistance	*ARR-2*	23.33
Phenicol resistance	*floR*	23.33
Macrolide resistance	*mph(A)*	23.33
Fosfomycin resistance	*fosA3*	23.33

## Data Availability

The original contributions presented in this study are included in the article/Supplementary Material. Further inquiries can be directed to the corresponding authors.

## Supplementary Materials

The following supporting information can be downloaded at: https://www.mdpi.com/article/10.3390/microorganisms14081767/s1, Figure S1: Heatmap of virulence genes identified in the *Salmonella* genomes. Each column represents an isolate, and each row represents a virulence gene. The color intensity indicates the sequence coverage of each virulence gene in each isolate. Table S1: Antimicrobial resistance genes and phenotype.
